# Self-reported diabetes is associated with self-management behaviour: a cohort study

**DOI:** 10.1186/1472-6963-8-142

**Published:** 2008-07-07

**Authors:** Baiju R Shah, Douglas G Manuel

**Affiliations:** 1 Institute for Clinical Evaluative Sciences, Toronto, Canada; 2 Departments of Medicine and Health Policy, Management and Evaluation, University of Toronto, Toronto, Canada; 3 Department of Medicine, Sunnybrook Health Sciences Centre, Toronto, Canada; 4 Department of Public Health Sciences, University of Toronto, Toronto, Canada

## Abstract

**Background:**

The purposes of this cohort study were to establish how frequently people with physician-diagnosed diabetes self-reported the disease, to determine factors associated with self-reporting of diabetes, and to evaluate subsequent differences in self-management behaviour, health care utilisation and clinical outcomes between people who do and do not report their disease.

**Methods:**

We used a registry of physician-diagnosed diabetes as a reference standard. We studied respondents to a 2000/01 population-based health survey who were in the registry (n = 1,812), and we determined the proportion who reported having diabetes during the survey. Baseline factors associated with self-report and subsequent behavioural, utilisation and clinical differences between those who did and did not self-report were defined from the survey responses and from linkage with administrative data sources.

**Results:**

Only 75% of people with physician-diagnosed diabetes reported having the disease. People who did self-report were more likely to be male, to live in rural areas, to have longer disease duration and to have received specialist physician care. People who did not report having diabetes in the survey were markedly less likely to perform capillary blood glucose monitoring in the subsequent two years (OR 0.05, 95% CI 0.02 to 0.08). They were also less likely to receive specialist physician care (OR 0.55, 95% CI 0.37 to 0.86), and were less likely to require hospital care for hypo- or hyperglycaemia (OR 0.09, 95% CI 0.01 to 0.28).

**Conclusion:**

Many people with physician-diagnosed diabetes do not report having the disease, but most demographic and clinical features do not distinguish these individuals. These individuals are much less likely to perform capillary glucose monitoring, suggesting that their diabetes self-management is inadequate. Clinicians may be able to use the absence of glucose monitoring as a screening tool to identify people needing a detailed evaluation of their disease knowledge.

## Background

Chronic disease care in the ambulatory setting is placing increasing emphasis on patient self-management. [1,2] Diabetes care is an example of this approach, as people with the disease are involved in many aspects of their care, including diet, exercise and other lifestyle interventions, self-monitoring of blood glucose levels, regular foot care, medication adherence and adjustment of insulin doses. Providing knowledge and skills to self-manage diabetes leads to small to moderate short-term improvements in both glycaemic and blood pressure control. [3,4]

To self-report having diabetes, a person must have at least some cognitive knowledge of the disease. They must recognize the term "diabetes" and associate the term with themselves. Most people who self-report having the condition likely associate diabetes as a risk to their health, although they may also associate diabetes with some positive aspects of their health, such as empowerment to modify their health risk, or membership in a group of similar people. However, not all people who are diagnosed with diabetes will self-report that they have the disease. People may not self-report having diabetes for several reasons: they may never have been properly informed about the diagnosis, they may not understand the term, they may disagree with the diagnosis, they may believe the disease is "cured" because they are managing it appropriately, or they may be hiding the diagnosis because of stigma about diabetes. Regardless of the reasons for not self-reporting diabetes, these people may be less likely to participate in disease self-management and hence may not achieve the best possible health outcomes.

Previous studies have reported that between one-quarter and one-half of people with diagnosed diabetes deny having it on health questionnaires. [5-8] Middle-aged adults were less likely to self-report having diabetes than younger adults or the elderly. [5,9] Some studies reported that women were more accurate, [10] while others suggested that men were. [5,9] Higher education levels resulted in greater rates of self-report. [6,10,11] Patients who do not self-report having diabetes have been found to have worse glycaemic control, [12] but otherwise the clinical consequences have not been studied.

We hypothesized that demographic factors (such as age and education) and clinical factors (such as other illnesses and physician utilisation) would be associated with self-reported diabetes in people with physician-diagnosed diabetes. We also hypothesized that people with diabetes who did not report having the disease would subsequently participate less in self-management behaviours, have reduced health care utilisation, and would therefore have worse clinical outcomes than those who did report their disease.

## Methods

The study base was all community-dwelling non-institutionalised residents of Ontario, Canada with physician-diagnosed diabetes in 2000/01, aged 20 years or older. The study was approved by the institutional review board of Sunnybrook Health Sciences Centre.

### Data sources

The study was conducted using a population-based disease registry, a population health survey, and health administrative data sources. Individuals were linked between all data sources using their encrypted unique health card number.

Physician-diagnosed diabetes was determined from the Ontario Diabetes Database (ODD), a registry of people with diabetes (excluding gestational diabetes) created from administrative data sources. [13] Diabetes is defined based on physician service claims and hospitalisation records bearing a diagnosis code for diabetes, [14] and the date of diagnosis is determined from the earliest such record. When compared against primary care chart review, the ODD was found to have at least 97% specificity. [13]

Self-reported diabetes was determined from the Canadian Community Health Survey cycle 1.1 (CCHS). The CCHS provided a cross-sectional estimate of health determinants, health status and health care utilisation in the Canadian population, and was conducted between September 2000 and November 2001 by Statistics Canada. The target population of the survey included household residents across Canada, excluding populations living on aboriginal reserves, military bases and certain remote areas. In Ontario, the sample size was 39,278 respondents, which represented a response rate of 82.0 percent. Of them, 32,848 respondents (83.6 percent) consented to and were successfully linked to administrative data using their encrypted health card number.

Other linked data sources used in this study included: 1) the discharge abstracts database, which provides detailed information on all hospitalisations; 2) the physician service claims database, which includes billing claims for consultation, visits and assessments by physicians; and 3) the drug prescription database, which lists prescriptions provided under the provincial formulary for all residents aged ≥65 years. Because of the government-funded universal health care system in Ontario, these data are comprehensive (covering all Ontario residents) and complete (covering virtually all delivered care across settings and across time).

### Patient selection

Adults surveyed in the CCHS who were also recorded in the ODD as having been diagnosed with diabetes by a physician at least six months before their CCHS interview date were selected. The lag allowed time for individuals to receive and understand the diagnosis of diabetes from their physicians. Self-reported diabetes was defined based on the answer to the CCHS question "Do you have diabetes?"

### Variable definitions

Several factors which could be associated with self-reported diabetes were defined from the CCHS, ODD and other administrative data. These baseline factors were determined as of the date of the CCHS interview. Sociodemographic variables were sex, age, marital status, immigration status, race, rural residence, education level and neighbourhood income level. Health status variables were duration of diabetes, number of other chronic medical conditions, microvascular disease (history of retinal photocoagulation) and macrovascular disease (history of hospitalisation for coronary disease, stroke, non-traumatic lower extremity amputation). Health care utilisation variables were ambulatory care visits with family physicians and diabetes specialist physicians in the year before the CCHS interview.

Subsequent self-management behaviour was assessed by examining whether people filled at least one prescription for capillary glucose test strips within two years after their CCHS interview date. Canadian practice guidelines recommend capillary glucose monitoring for virtually all people with diabetes. [15] Because only people aged ≥65 years are eligible for the provincially-funded drug benefits program, these data were not available for younger people, so they were excluded from this analysis. The measures of subsequent health care utilisation (also measured within two years after the person's CCHS interview date) were: at least one visit for ophthalmological screening with an eye care professional, and at least one ambulatory visit for diabetes specialist care. The time from the CCHS interview to the first occurrence of several clinical outcomes (hospitalisation or emergency department visit for hypo- or hyperglycaemia, hospitalisation for soft-tissue infections or gangrene, hospitalisation for myocardial infarction, and death) were also determined.

### Statistical analysis

To evaluate the factors associated with self-reported diabetes, logistic regression was used with self-reported diabetes as the dependent variable and the baseline factors as independent variables. Both bivariate and multivariate models were built using all factors.

To determine whether subsequent self-management behaviour and health care utilisation were different between people who did and did not self-report having diabetes, we used logistic regression adjusting for all baseline factors. The impact on subsequent clinical outcomes was assessed using Cox proportional hazards regression, adjusting for all baseline factors plus two additional variables from the CCHS: self-perceived health and smoking status. Censoring was at death or the end of follow-up (March 31, 2005). In all models, individuals were weighted based on their sampling weight in the CCHS, and bootstrapping methods were used to determine 95% confidence intervals around all estimates.

## Results

There were 1,812 people with physician-diagnosed diabetes (according to the reference standard definition of diabetes) who completed the CCHS. Of them, 1,356 (75%) self-reported having diabetes, while 456 (25%) did not.

Bivariate and multivariate associations between baseline factors and self-reported diabetes, including odds ratios with 95% confidence intervals, are presented in Table 1. People who were male, lived in rural areas, with longer diabetes duration and who saw diabetes specialists were more likely to report that they had the disease. Other factors were not statistically significantly associated with self-reported diabetes.

**Table 1:** Bivariate and multivariate associations of baseline factors with self-reported diabetes.

**Predictor**		**Frequency of self-reported diabetes (%)**	**Weighted bivariate OR (95% CI)**	**Weighted multivariate OR (95% CI)**
Sex	Male	681/886 (77)	1.00 ref	1.00 ref
	Female	675/926 (73)	0.72 (0.52–0.98)	0.63 (0.44–0.87)
Age	18–44	150/212 (71)	0.70 (0.44–1.12)	0.93 (0.54–1.73)
	45–64	490/641 (76)	1.00 (0.69–1.44)	1.22 (0.81–1.86)
	65 +	716/959 (75)	1.00 ref	1.00 ref
Marital status	Married	750/1012 (74)	1.00 ref	1.00 ref
	Widowed/Divorced	478/631 (76)	1.26 (0.90–1.84)	1.31 (0.89–2.00)
	Single	128/169 (76)	1.17 (0.70–2.10)	1.24 (0.68–2.32)
Immigrant	No	1056/1393 (76)	1.00 ref	1.00 ref
	Yes	300/419 (72)	0.76 (0.53–1.08)	0.80 (0.52–1.20)
Race	White	1239/1641 (76)	1.00 ref	1.00 ref
	Non-White	117/171 (68)	0.82 (0.51–1.28)	1.03 (0.60–1.72)
Rural residence	No	1072/1442 (74)	1.00 ref	1.00 ref
	Yes	284/370 (77)	1.52 (1.10–2.22)	1.59 (1.06–2.41)
Education level	Less than high school	603/790 (76)	1.00 ref	1.00 ref
	Graduated high school	232/310 (75)	0.94 (0.58–1.52)	1.04 (0.64–1.72)
	Some post-secondary	85/111 (77)	1.02 (0.57–1.99)	0.91 (0.46–1.81)
	Graduated post-secondary	436/601 (73)	0.93 (0.64–1.33)	0.97 (0.63–1.49)
Income quintile	Lowest	337/434 (78)	1.00 ref	1.00 ref
	2	305/421 (72)	0.65 (0.40–1.09)	0.63 (0.36–1.08)
	3	272/364 (75)	0.74 (0.44–1.20)	0.78 (0.45–1.30)
	4	235/319 (74)	0.75 (0.44–1.27)	0.78 (0.45–1.35)
	Highest	207/274 (76)	0.79 (0.46–1.29)	0.81 (0.45–1.41)
Diabetes duration	6 months to 2 years	164/269 (61)	1.00 ref	1.00 ref
	2 to 5 years	335/479 (70)	1.56 (0.96–2.49)	1.78 (1.07–2.89)
	5+ years	857/1064 (81)	2.69 (1.62–4.13)	2.82 (1.66–4.43)
Other chronic conditions	0	756/1031 (73)	1.00 ref	1.00 ref
	1	405/529 (77)	1.40 (0.98–2.05)	1.19 (0.80–1.85)
	2 or more	195/252 (77)	1.19 (0.70–2.16)	1.01 (0.60–1.91)
Microvascular disease	No	1316/1769 (74)	1.00 ref	1.00 ref
	Yes	40/43 (93)	1.85 (0.54–8)	1.83 (0.77–8)
Macrovascular disease	No	1240/1674 (74)	1.00 ref	1.00 ref
	Yes	116/138 (84)	1.22 (0.69–2.57)	0.81 (0.41–1.84)
Family physician visits	0 to 6	585/812 (72)	1.00 ref	1.00 ref
	7 to 12	328/422 (78)	1.25 (0.86–1.87)	1.31 (0.87–2.09)
	13 or more	443/578 (77)	1.24 (0.85–1.91)	1.18 (0.76–2.01)
Specialist visits	No	917/1271 (72)	1.00 ref	1.00 ref
	Yes	439/541 (81)	1.90 (1.34–2.79)	1.95 (1.34–2.96)

The subsequent differences between those who did and did not report having diabetes are presented in Table 2. People who did not report having diabetes were markedly less likely to perform capillary blood glucose monitoring than those who did. They were also less likely to have specialist physician care, but ophthalmologic screening did not differ. Many of the clinical events were uncommon. Nonetheless, only four of the people who had a hospitalisation or emergency department visit for hypo- or hyperglycaemia had not reported having diabetes in the CCHS; hence, the adjusted hazard ratio for these events was highly statistically significant. Other clinical outcomes did not differ between groups.

**Table 2:** Subsequent differences between subjects who did and did not report having diabetes.

**Measure**		
**Self-management behavior**	**Overall frequency**	Weighted adjusted OR (95% CI)*

Capillary blood glucose monitoring ^†^	63.1%	0.05 (0.02–0.08)

**Health care utilization**	**Overall frequency**	Weighted adjusted OR (95% CI) *

Ophthalmologic exam	74.3%	0.82 (0.56–1.22)
Specialist visit	41.9%	0.55 (0.37–0.86)

**Clinical outcomes**	**Overall event occurrence**	Weighted adjusted HR (95% CI) *

Hypo- or hyperglycemia	4.8%	0.09 (0.01–0.28)
Soft tissue infection or gangrene	3.1%	0.47 (0.11–1.28)
Myocardial infarction	5.5%	0.92 (0.32–1.50)
Death	13.9%	1.02 (0.67–1.59)

* Subjects who self-reported having diabetes are the reference group.

^† ^This measure was determined only in subjects ≥65 years (n = 959).

## Discussion

More than one in four people with physician-diagnosed diabetes did not self-report having the disease. This frequency is similar to that reported in previous studies. [5-8] Our findings also showed that people who did not self-report having diabetes were markedly less likely to perform capillary blood glucose monitoring, an example of the many self-management behaviours expected of people with diabetes. Since self-care is the cornerstone of diabetes management, a substantial portion of the diabetic population may be inadequately managed. The lack of self-identification as being diagnosed with diabetes may occur for many reasons, because of a lack of awareness, belief in or acceptance of the diagnosis. It might also occur because physicians simply did not inform their patients of the diagnosis, or used euphemisms rather than the word "diabetes". Individuals who do not self-identify as having diabetes may not be aware of the attendant risks to their health. They may also be less likely to respond to public education messages about diabetes, and if they do not report having the disease to their physicians and other health care providers, diabetes may not be taken into account in treatment plans for their other health conditions.

To overcome this barrier to care and to encourage patient self-management, clinicians may want to identify people who do not self-report having diabetes. The baseline factors most strongly associated with self-reported diabetes were longer duration of diabetes and specialist care for the disease. These are perhaps not surprising, as those who have had diabetes for more time and who are attending a specialist clinic to treat it should be more likely to disclose having the disease. However, the other factors that might have been expected to be associated with self-report (such as education, income, ethnicity or the presence of other chronic diseases) in fact were not, so clinicians cannot rely on these factors to identify individuals who do not report having the disease. In fact, capillary glucose monitoring may be a marker to identify such people. Monitoring may reflect individuals' knowledge and understanding of diabetes and their engagement in its treatment, so clinicians may be able to use the absence of this self-management behaviour as a screening tool to identify individuals needing a more detailed assessment of their comprehension of diabetes and its management.

Unlike previous studies examining self-reported diabetes, this study included a large number of people and was population-based, and hence had greater power to detect factors associated with self-report and differences in care between those who did and did not report having diabetes. An important limitation, however, is the absence of important clinical and behavioural measures that could be more influenced by the failure to self-report diabetes than the health service utilisation and outcomes measures used. In addition, a more detailed survey would be needed to discern the potential psychological and behavioural reasons why some people may fail to self-report diabetes. The reference standard definition of diabetes we used for physician-diagnosed diabetes was an administrative-data derived disease registry. While a validation study suggested excellent specificity (≥97%), false positives will occur. [13] Such people would be categorized as failing to self-report diabetes, even though they genuinely do not have the disease. Although the registry excludes gestational diabetes, some such patients may have been inadvertently captured, which might explain the apparently lower frequency of self-report among women.

Differences in health service utilisation between those who do and do not self-report having diabetes have not been evaluated previously. The study found that ophthalmologic screening examinations occurred whether or not people reported having diabetes, but people who did not report diabetes were less likely to receive specialist physician care for diabetes in the following two years, even after adjusting for baseline specialist utilisation and diabetes severity. This finding suggests that patients may need to advocate on their own behalf for referral to diabetes specialists, which those not reporting diabetes may be less likely to do. It also suggests that primary care physicians are far more likely than specialists to have to identify people who do not report having diabetes and to overcome this potential barrier to care.

The failure to self-identify disease has been studied in other diseases, where important consequences have been noted. People who did not report having hypercholesterolemia made fewer dietary changes than those who did, [16] corroborating the effect of self-report on self-management behaviour. Denial of illness predicted worse survival in colon cancer. [17] Following acute myocardial infarction or coronary artery by-pass surgery, patients who did not acknowledge the seriousness of their illness were more noncompliant with treatment and spent more days readmitted to hospital during the following year. [18] In contrast, several studies have documented that women with breast cancer who did not report having the disease had better long-term survival than those who acknowledged the disease, even when controlling for tumour staging, treatments performed and other prognostic factors. [19-21]

## Conclusion

A large proportion of people with physician-diagnosed diabetes do not self-report that they have the disease, which may in part reflect physicians' failure to appropriately convey the diagnosis. These individuals are less likely to perform capillary blood glucose monitoring, a marker of self-management behaviour. Since self-care is a foundation of the treatment for chronic diseases, these individuals' diabetes may not be ideally managed. Sociodemographic factors do not help clinicians identify people do not report their disease. Clinicians, particularly primary care practitioners, must identify such individuals to ensure that they are aware of and acknowledge their diagnosis, and are adequately educated about its optimal management.

## Abbreviations

CCHS: Canadian Community Health Survey; ODD: Ontario Diabetes Database.

## Competing interests

The authors declare that they have no competing interests.

## Authors' contributions

BRS conceived of the study, participated in the design of the study and wrote the manuscript. DGM participated in the design of the study. Both authors read and approved the final manuscript.

## Pre-publication history

The pre-publication history for this paper can be accessed here:


